# Robust and durable response to first-line treatment of pembrolizumab combined with chemotherapy in two patients with metastatic thymic squamous cell carcinoma: Case report

**DOI:** 10.3389/fimmu.2022.941092

**Published:** 2022-08-02

**Authors:** Cui Chen, Peng Sun, Jianting Long

**Affiliations:** ^1^ Department of Oncology, The First Affiliated Hospital, Sun Yat-Sen University, Guangzhou, China; ^2^ Department of Medical Oncology, Sun Yat-Sen University Cancer Center, State Key Laboratory of Oncology in South China, Collaborative Innovation Center for Cancer Medicine, Guangzhou, China

**Keywords:** metastatic thymic carcinoma, first-line treatment, pembrolizumab, durable response, case report

## Abstract

Thymic carcinoma is a rare and aggressive disease with poor outcome. There is no established treatment regimen for advanced thymic carcinoma. While the efficacy of pembrolizumab was proved to be promising, as a single agent, in patients with refractory/recurrent thymic carcinoma that progressed after chemotherapy, the efficacy and safety of combination of pembrolizumab and chemotherapy as front-line treatment in metastatic thymic carcinoma have not been explored yet. Herein, we report the first two cases of metastatic thymic squamous cell carcinoma receiving the combined approaches of pembrolizumab and chemotherapy as first-line treatment. Of the two patients, one had a complete radiological response of mediastinal masses with sustained remission over 3 years, and the other one with widespread disease had a good partial response over 20 months and achieved no evidence of disease radiologically after undergoing percutaneous radiofrequency ablation for residual liver metastases. Next-generation sequencing (NGS) showed low tumor mutation burden and MSS in both patients. Immunohistochemistry analysis of the tumor showed high PD-L1 expression in patient 1 and low PD-L1 expression in patient 2. Pembrolizumab combined with chemotherapy may be an attractive strategy for the first-line treatment of metastatic thymic carcinoma and thus warrants further evaluation.

## Introduction

Thymic carcinoma is a very rare and aggressive cancer with poor outcomes and limited treatment options for advanced stages of the disease (1–3). Chemotherapy remains the standard treatment for metastatic thymic carcinoma. However, chemotherapy, mainly platinum-based regimen, has limited clinical activity with response rates of 20%–30% reported in previous clinical trials in advanced thymic carcinomas (4, 5). More effective treatments are needed for this rare disease.

The promising efficacy of pembrolizumab has been shown in first-line treatment in many solid tumors (6–11). Several phase II studies have shown that pembrolizumab may become a promising treatment option in patients with advanced thymic carcinoma (12, 13). Theoretically, chemotherapy can promote the release of tumor-associated neoantigens and improve the effect of immune checkpoint inhibitors (ICIs) (14). The combination of immunotherapy with cytotoxic agents has shown encouraging antitumor activities in multiple tumor types (6–9). However, there is a lack of clinical trials to support the first-line treatment with combination of pembrolizumab and chemotherapy in metastatic thymic carcinoma due to the rarity of this tumor.

The effect of combination of ICIs and chemotherapy as front-line treatment in thymic carcinoma remains undetermined. Herein, we report two cases of metastatic thymic carcinoma that exhibited a robust and durable response to first-line treatment of pembrolizumab, a PD-1 blockade, in combination with chemotherapy.

## Case presentation

**Figure 1:**
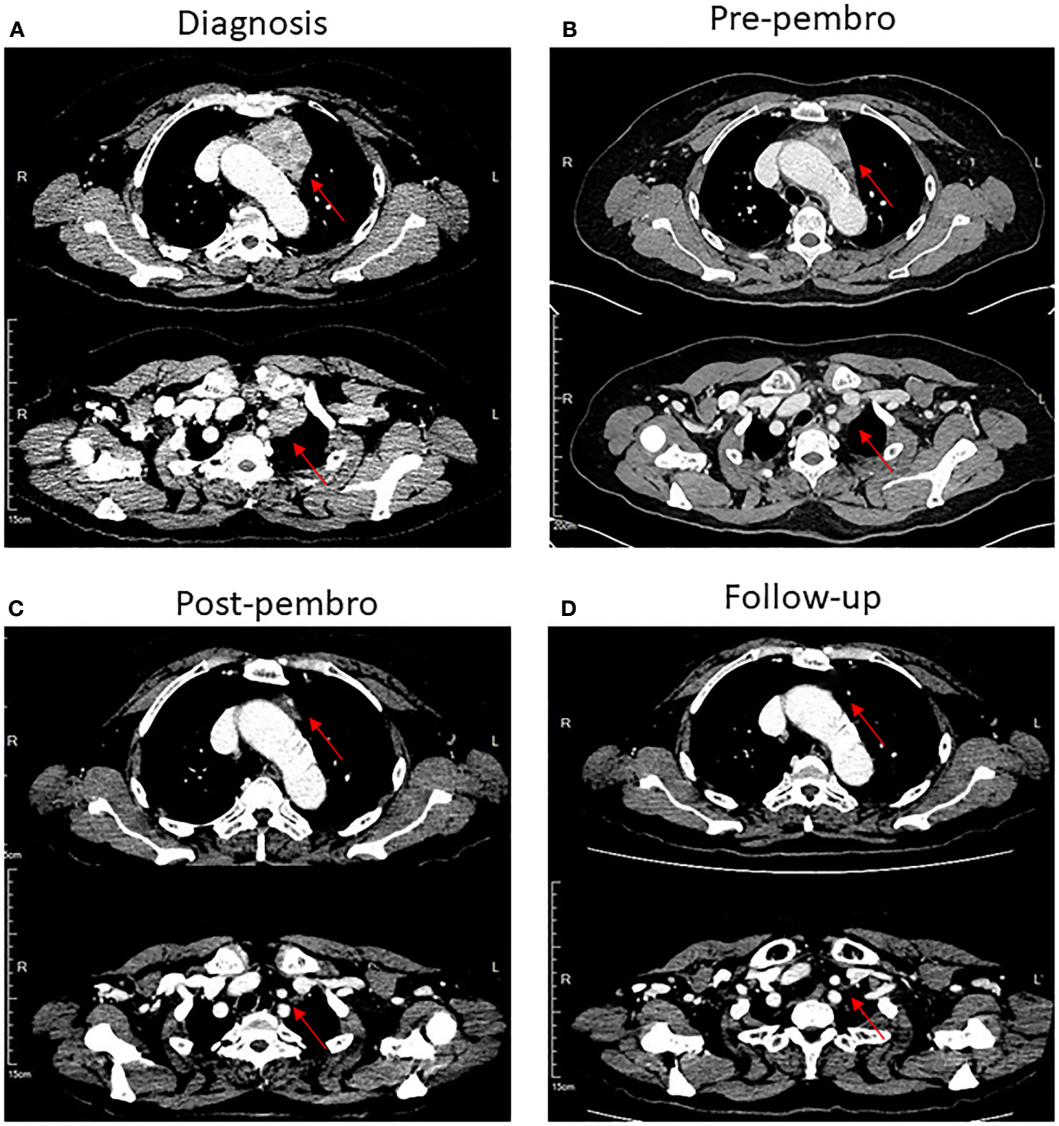
CT scans of the chest showed that patient 1 had a complete radiological response of mediastinal masses after treatment with pembrolizumab in combination with chemotherapy. **(A)** Mediastinal masses at diagnosis, lesion has been marked with red arrows. **(B)** Mediastinal masses before treatment with pembrolizumab combined with chemotherapy. **(C)** Mediastinal masses are markedly improved after treatment with four cycles of pembrolizumab combined with chemotherapy. **(D)** Disappearance of mediastinal masses in follow-up.

Patient 1, a 63-year-old woman, presented with chest pain and disability in the right leg in January 2019. She was unable to walk at admission, with a performance status (PS) score of two points. She had no significant past or family medical histories. PET-CT and CT scans showed an abnormal shadow in the anterior mediastinum, mediastinal lymph node metastasis, and right femoral neck metastasis with pathological fracture of the femur (**Figure 1A**). A CT-guided percutaneous mediastinal mass biopsy revealed low differentiated squamous cell carcinoma arising from the thymus, with CD117(+), P40(+), CK focally (+), WT1 focally (+), CK7(+), CD5(-), TTF-1(-), NapsinA(-), and SALL4(-) (**Supplementary Figure 1A**). Given these findings, her final diagnosis was thymic squamous cell carcinoma with mediastinal lymph nodes and right femoral neck metastases (Masaoka stage IVb).

The patient received one cycle of docetaxel (75 mg/m^2^) plus cisplatin (75 mg/m^2^). As the patient suffered from serious gastrointestinal side effects after the first cycle of chemotherapy, the regimen was thus switched to docetaxel (75 mg/m^2^) plus carboplatin (AUC = 5). After two cycles of chemotherapy, tumor size in the anterior mediastinum was reduced (**Figure 1B**).

**Figure 2:**
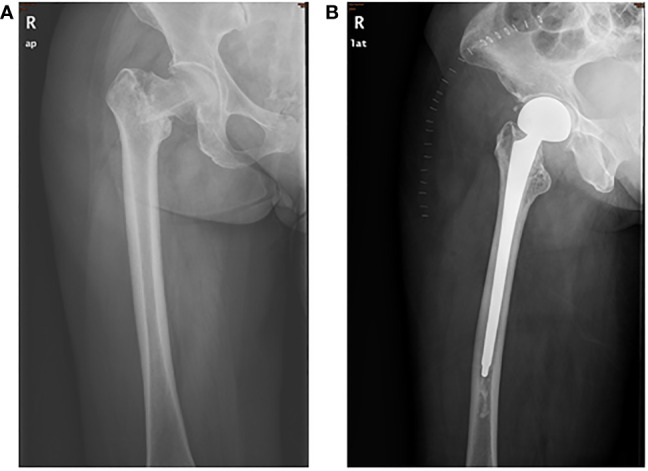
X‐ray films of the right femur **(A)** before surgery and **(B)** after surgery.

Next-generation sequencing (NGS) of her tumor showed a low tumor mutation burden (TMB) with 3.6 mutations/megabase and microsatellite stable (MSS) status. There were no clearly druggable driver mutations and no potential biomarkers of increased risk of hyperprogression after immunotherapy. Immunohistochemistry analysis of the tumor demonstrated 85% PD-L1 expression on tumor cells. PD-L1 tumor cell proportion score (TPS) was 85% (clone SP142, Ventana, **Supplementary Figure 1B**). The patient has no history of autoimmune disease and PS score was improved after prior chemotherapy. Based on these and the patient’s strong willingness, the patient was enrolled in PD-1 inhibitor therapy. She received a total of four cycles of the aforementioned chemotherapy along with 200 mg of pembrolizumab as a fixed dose at day 1 every 3 weeks. After four cycles of the combination therapy, her tumor in the mediastinum almost disappeared (**Figure 1C**). Subsequently, she received proximal femur tumor excision and artificial femoral head replacement in June 2019 (**Figure 2**). Thereafter, the clinical symptoms were totally relieved. Postoperative pathology of proximal femur tumor excision revealed no significant cytologic atypia but densely infiltrating lymphocytes. Afterwards, the patient underwent pembrolizumab maintenance treatment of 200 mg every 3 weeks for 1 year and mediastinal tumor masses had disappeared completely (**Figure 1D**). The patient achieved excellent performance status and quality of life with no evidence of disease. Overall, no serious adverse events were observed. The main adverse reactions were mild fatigue, leukopenia, and alopecia. Treatment was discontinued because the patient developed osteonecrosis of the jaw due to long-term usage of bisphosphonate. However, the patient’s continuous remission is still ongoing at the time of this report and the duration of response has been achieved over 3 years. The treatment timeline is shown in **Supplementary Figure 2**.

Patient 2, a 68-year-old woman, was diagnosed as having locally advanced thymic carcinoma and underwent surgery in November 2018. The pathology revealed thymic poorly differentiated squamous cell carcinoma. Subsequently, she received concurrent radio-chemotherapy, including adjuvant radiotherapy and six cycles of chemotherapy of nab-paclitaxel plus cisplatin.

**Figure 3:**
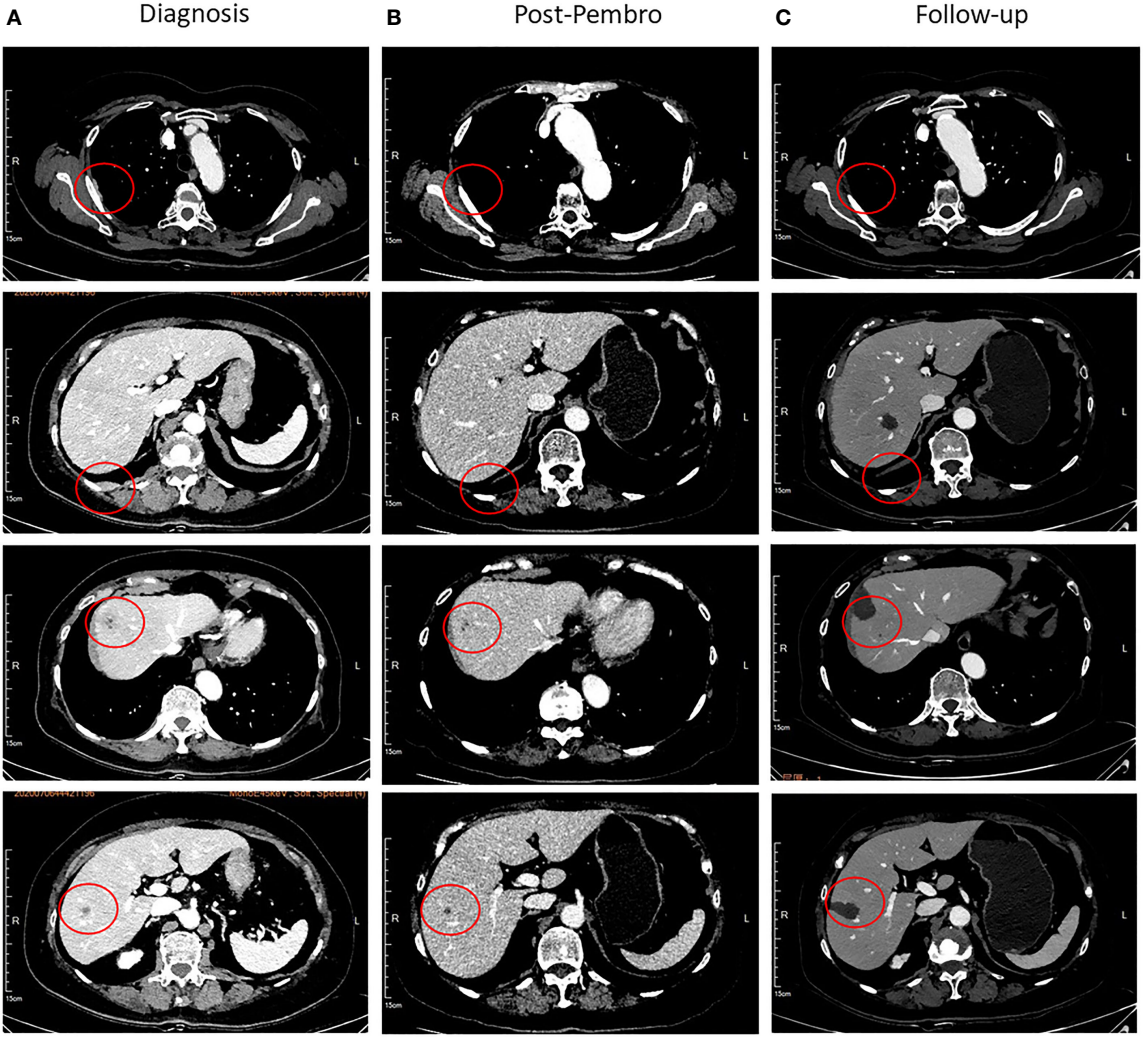
Representative images of the CT scan. **(A)** The pleura and liver lesions at diagnosis, lesion has been marked with red circles. **(B)** Disappearance of pleura lesions and shrinkage of liver lesions after four cycles of chemotherapy combined with pembrolizumab. **(C)** Follow-up after radiofrequency ablation.

**Figure 4:**
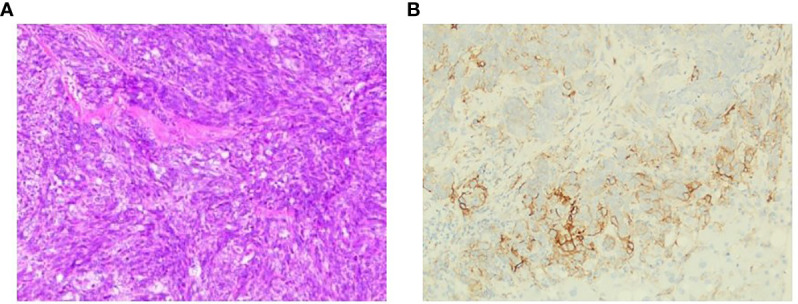
Representative micrographs. **(A)** Liver lesion biopsy, squamous cell carcinoma. **(B)** PD-L1 expression, 5% PD-L1 expression on tumor cells.

However, regular outpatient follow-up CT scan examination in July 2020 found multiple pleural nodules and liver lesions (**Figure 3A**). Subsequent PET-CT showed multiple distant metastases to liver and pleura. Additionally, ultrasound-guided percutaneous liver lesion biopsy was carried out, showing squamous cell carcinoma arising from the thymus, with CD117(+), P40(+), P63(+), CK(+), CD5 focally (+), CK5/6(+), Hepatocyte(-), and Glypican-3(-) (**Figure 4A**). Taken together, the patient was diagnosed as having multiple metastatic squamous cell carcinoma in liver and pleura after a year and a half. In addition, she had histories of type 2 diabetes mellitus and high blood pressure for years. She had no other significant past or family medical histories.

NGS of her tumor showed a low TMB with 4.21 mutations/megabase and MSS status. Deleterious alterations occurred in the FGFR3 gene (c.746C>G). No immune-related negative predictor was observed. Upon immunohistochemical analysis, the tumor demonstrated 5% positivity for PD-L1 expression *via* clone SP142 antibody [PD-L1 tumor cell proportion score (TPS) = 5%, **Figure 4B**]. The combination of pembrolizumab and chemotherapy regimen was performed for six cycles. A fixed dose of pembrolizumab (200 mg) was given at day 1 every 3 weeks. Chemotherapy regimen is nab-paclitaxel (260 mg/m^2^) plus carboplatin (AUC = 5) in the first cycle, and carboplatin was eliminated from the second to sixth cycle due to many concomitant diseases including high blood pressure, cardiovascular disease, and diabetes. After four cycles of the combination therapy, her metastatic pleura nodules disappeared and liver lesions were strikingly reduced with an evaluation of partial response by RECIST version 1.1 (**Figure 3B**). After six cycles of combination regimen, the patient received pembrolizumab maintenance treatment of 200 mg every 3 weeks. By virtue of study results that residual tumor cells may induce T-cell depletion (15), the patient underwent percutaneous radiofrequency ablation for residual liver metastases based on multidisciplinary tumor board review in January and March 2021. Thereafter, there was no evidence of disease radiologically. No serious adverse events were observed during these treatments. She continued to have no evidence of disease with excellent performance status and quality of life so far (**Figure 3C**). The duration of response has been achieved over 20 months. The treatment timeline is shown in **Supplementary Figure 3**.

## Discussion

There are many reports about exceptional and durable responses to ICIs in solid tumors. To our knowledge, however, this is the first report of pembrolizumab combined with chemotherapy in the first-line treatment of metastatic thymic carcinoma gaining durable response and good tolerance. Most importantly, both patients with widespread disease achieved no evidence of disease radiologically after multidisciplinary treatment.

Chemotherapy is generally offered as the initial treatment for patients with metastatic or recurrent thymic carcinoma (3). However, chemotherapy yielded limited benefits. In a phase II study of carboplatin and paclitaxel in advanced thymic carcinoma, the response rate was only 21.7% and no CRs were observed. Median PFS was only 5 months (4). Currently, no standard regimens were established for the treatment of patients with metastatic or recurrent thymic carcinoma. A new treatment strategy was warranted for this kind of patients.

In the last few years, the discovery of ICI has revolutionized the treatment of many cancers (16). Compared with cytotoxic chemotherapeutic agents, ICI has shown the ability to reach long-term remission in a subset of patients (17). Several studies show that thymic carcinomas have a high expression of PD-L1, which is more likely to benefit from ICI (18, 19). Pembrolizumab is a highly selective anti-PD-1 humanized monoclonal antibody that blocks the PD-1 pathway, reverses T-cell suppression, and induces antitumor responses (20). Several phase II studies showed that pembrolizumab has encouraging antitumor activity in patients with advanced thymic carcinoma and several patients achieved durable response (12, 13). Giaccone et al. reported that the overall response rate of pembrolizumab monotherapy in advanced thymic cancer was 22.5% and the duration of response was approximately 3 years in an update of the phase II study (21). As for advanced squamous cell carcinoma, chemotherapy alone yielded limited response and short duration of response. Combination of ICIs and chemotherapy as an initial treatment may result in more initial remissions and durable responses. Theoretically, there are some synergetic effects when applying the combination of ICIs and chemotherapy. Many researchers have investigated the possible mechanisms of the synergistic antitumor effect of combination therapy so far, of which, taxane and platinum derivatives attract the most attention. Studies show that systemic chemotherapy may stimulate anti-cancer immune effectors or inhibit immunosuppressive factors, enhancing immune recognition and T-cell responsiveness (22–26). In patients with advanced squamous cell carcinoma of the lung, a phase III study, KEYNOTE-407, showed that the front-line addition of pembrolizumab to platinum-doublet chemotherapy had dramatically improved response rate (58.4% vs. 35.0%) and median overall survival (17.1 months vs. 11.6 months) (6, 7). Another phase III study, KEYNOTE-048, showed that pembrolizumab with chemotherapy improved duration of response (CPS≥1: 6.7 months vs. 4.3 months) and overall survival (13.0 months vs. 10.7 months) versus cetuximab with chemotherapy in head and neck squamous cell carcinoma (9). Furthermore, it has been proven that the standard dose of pembrolizumab can be safely and effectively combined with chemotherapy in a wide range of solid tumors, such as advanced non-small cell lung cancer (NSCLC), head and neck squamous cell carcinoma, gastric cancer, and additional forms of cancer (6, 7, 9, 27). These results thus have provided grounds for subjecting our patients to pembrolizumab combined with chemotherapy. In our study, the two patients gained robust and durable response. One patient had complete response with sustained remission over 3 years. The other patient had good partial response over 20 months and achieved no evidence of disease radiologically after undergoing percutaneous radiofrequency ablation for her residual liver metastases. Hence, exploring the efficacy of combining immunotherapy and chemotherapy as an initial treatment in metastatic thymic cancer is promising.

Another issue that needs to be addressed is how to identify the potential population that would benefit from pembrolizumab. Currently, three factors are often used to identify the patients more likely to respond to pembrolizumab, namely, PD-L1 expression on tumor cells, microsatellite instability (MSI), and TMB (28). We performed NGS and immunohistochemistry analysis of PD-L1 in these two patients. Both of the patients with low TMB and MSS status responded well to pembrolizumab, although high TMB and MSI were considered as predictive biomarkers for anti-PD-1 immunotherapy in many cancers. As for thymic cancer, there was no evidence that TMB and MSI had the potential to be an appropriate predictive marker. Furthermore, previous studies have shown that thymic tumors have the lowest TMB among all adult cancers (29). In practice, PD-L1 expression is commonly used to select the patients that are thought to benefit from immunotherapy (30). Recent studies reported that high expression of PD-L1 was observed in 36%–80% of thymic carcinomas (18, 19). Conflicting results were observed regarding the prognostic impact of PD-L1 expression level. However, high PD-L1 expression (≥50%) was associated with better ORR and PFS of pembrolizumab monotherapy in an exploratory analysis of the phase II studies (12, 13). Although PD-L1 expression is clinically validated as a predictive factor of response to pembrolizumab, especially when it is applied as a single agent, it is generally accepted that a clinically better outcome can also be achieved in a subpopulation with low PD-L1 expression, especially when pembrolizumab was applied together with chemotherapy. In the KEYNOTE-189 and KEYNOTE-407 trials, patients with less than 1% PD-L1 expression can also benefit from the combination of chemotherapy and immunotherapy (6, 7). Although patients with a high expression of PD-L1 may be more likely to benefit from pembrolizumab, the status of biopsy tissue is insufficient to capture the whole picture of tumor. In fact, in our study, patient 2 with weak PD-L1 expression responded well to the combination of pembrolizumab and chemotherapy as well. Overall, the treatment outcomes of our two cases suggest that the combination of pembrolizumab and chemotherapy may provide an effective therapeutic option for those thymic cancer patients with low values of immune predictive biomarkers.

Lastly, both patients tolerated immunotherapy well except for the fact that patient 1 developed osteonecrosis of the jaw. However, we did not consider this adverse event to be related to immunotherapy because the patient was also treated with zoledronic acid at that time. In contrast to thymoma, thymic cancer is known to lack immature T lymphocytes and may not induce autoimmune diseases (31). However, an increased risk of developing severe irAEs for thymic cancer was observed in a previous clinical trial (12). Thus, careful selection of patients and monitoring strategies are essential for treatment with PD-1 blockade.

## Conclusion

In this report, we have described two patients with metastatic thymic squamous cell carcinoma who have demonstrated robust and durable response to first-line treatment of pembrolizumab combined with chemotherapy. This strategy of pembrolizumab combined with chemotherapy may provide an effective therapeutic option for late-stage thymic cancer patients and thus warrants future evaluation in clinical trials.

## Data availability statement

The original contributions presented in the study are included in the article/**Supplementary material**. Further inquiries can be directed to the corresponding author.

## Ethics statement

The studies involving human participants were reviewed and approved by the Ethics Committee of The first Affiliated Hospital of Sun Yat Sen University (Guangzhou, China). The patients/participants provided their written informed consent to participate in this study. Written informed consent was obtained from the individual(s) for the publication of any potentially identifiable images or data included in this article.

## Author contributions

JL designed and supervised the study. CC and PS were in charge of manuscript drafting and data collection. All authors contributed to the article and approved the submitted version.

## Funding

This study was supported by grants from the Youth Funds of the Basic and Applied Basic Research Foundation of Guangdong Province (No.2020A1515110089) and the National Natural Science Foundation of China (nos.82103579).

## Conflict of interest

The authors declare that the research was conducted in the absence of any commercial or financial relationships that could be construed as a potential conflict of interest.

## Publisher’s note

All claims expressed in this article are solely those of the authors and do not necessarily represent those of their affiliated organizations, or those of the publisher, the editors and the reviewers. Any product that may be evaluated in this article, or claim that may be made by its manufacturer, is not guaranteed or endorsed by the publisher.

## Supplementary material

Supplementary Figure 1 Representative micrographs **(A)** mediastinal mass biopsy, thymic squamous cell carcinoma **(B)** PD-L1 expression, 85% PD-L1 expression on tumor cells.

Supplementary Figure 2 The treatment timeline of patient 1.

Supplementary Figure 3 The treatment timeline of patient 2.

## Abbreviations

ICIs, immune checkpoint inhibitors; NGS, next-generation sequencing; MSS, microsatellite stable; PS, performance status; TPS, tumor cell proportion score; TMB, tumor mutation burden; irAEs, immune-related adverse events; ORR, overall response rate; PFS, progression-free survival.
