# Supplementary figures and images for: Robust and durable response to first-line treatment of pembrolizumab combined with chemotherapy in two patients with metastatic thymic squamous cell carcinoma: Case report
Source: Front Immunol. 2022 Aug 2;13:941092. doi: 10.3389/fimmu.2022.941092 (PMC9378774; doi:10.3389/fimmu.2022.941092)

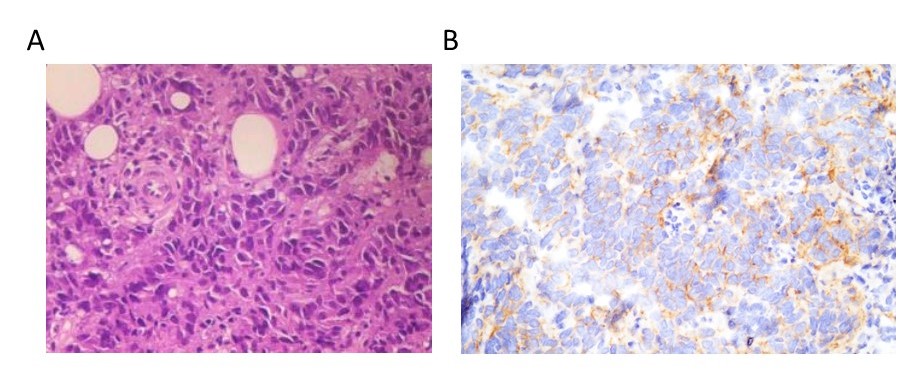

Supplement: Supplementary Figure 1 — Representative micrographs (A) mediastinal mass biopsy, thymic squamous cell carcinoma (B) PD-L1 expression, 85% PD-L1 expression on tumor cells. [file Image_1.jpeg]

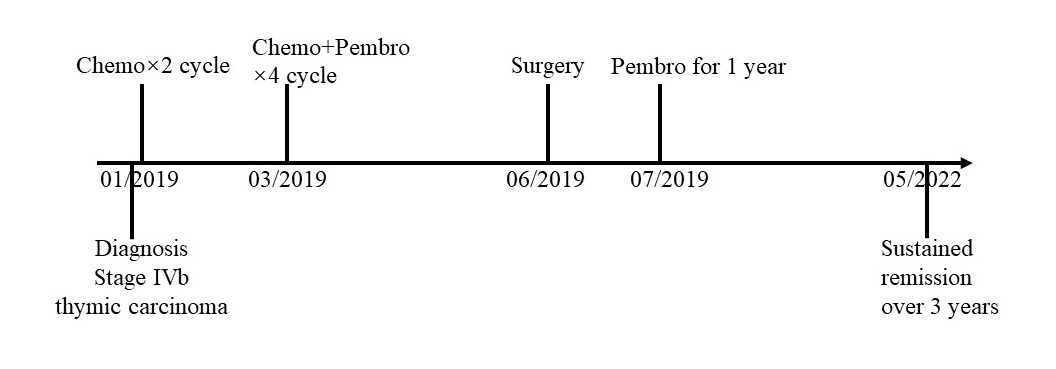

Supplement: Supplementary Figure 2 — The treatment timeline of patient 1. [file Image_2.jpeg]

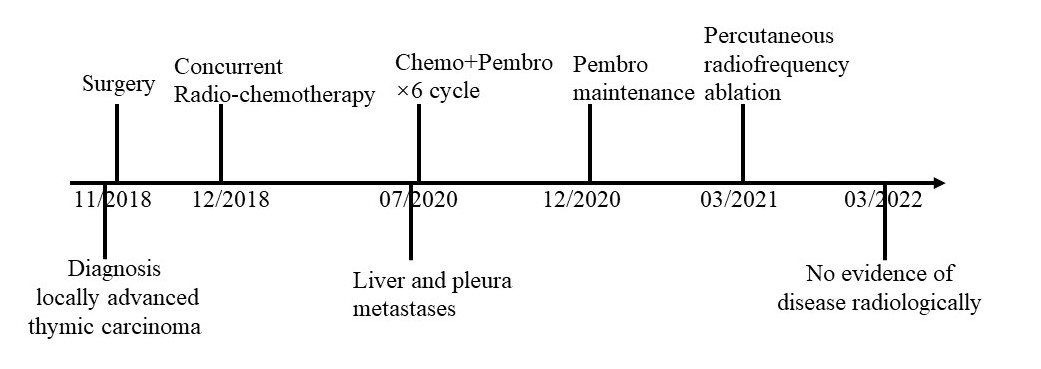

Supplement: Supplementary Figure 3 — The treatment timeline of patient 2. [file Image_3.jpeg]
